# Addition of nucleoside analogues to peg-IFNα-2a enhances virological response in chronic hepatitis B patients without early response to peg-IFNα-2a: a randomized controlled trial

**DOI:** 10.1186/s12876-017-0657-y

**Published:** 2017-08-30

**Authors:** Yan Xu, Xu Wang, Zhenhua Liu, Changyu Zhou, Wenqian Qi, Jian Jiao, Fan Yu, Honghua Guo, Ping Zhao, Jiangbin Wang

**Affiliations:** Department of Gastroenterology, China–Japan Union Hospital, Jilin University, Changchun, Jilin Province 130033 China

**Keywords:** Chronic hepatitis B, Interferon, Nucleoside analog, Virological response, Combination therapy

## Abstract

**Background:**

Current treatments for chronic hepatitis B (CHB) include pegylated interferon alpha (PEG-IFN-α) which is an immune modulator, and nucleos(t)ide analogs (NAs) which directly inhibit HBV DNA polymerase. With the limited efficacy of PEG-IFN-α and prolonged treatment periods associated with NAs, there is an urgent need for novel therapeutic strategies, especially for patients with a poor early response to anti-viral therapy.

**Methods:**

In this study, 178 patients with chronic hepatitis B (*n* = 131) and compensated (*n* = 47) HBV-induced cirrhosis were enrolled, 120 patients with HBeAg (+). All the patients were treated for 12 weeks with PEG-IFN-α. Among them, a total of 138 patients with a poor virological response after 12 weeks were treated for an additional 48 weeks with Peg-IFNα-2a (control) (*n* = 43), with Peg-IFNα-2a + entecavir (ETV) (*n* = 49), or Peg-IFNα-2a + adefovir dipivoxil (ADV) (*n* = 46), and were followed for 48 weeks after therapy. Early virological response was defined as undetectable HBV DNA after anti-viral therapy for 12 weeks. Sustained virological response (SVR) was defined as no change in therapeutic effectiveness after 6 months follow-up, and no recurrence.Therapeutic efficacy was determined by evaluating HBV DNA levels, serum and liver HBsAg levels, liver function tests and liver histology.

**Results:**

Patients in the Peg-IFNα-2a + ETV and Peg-IFNα-2a + ADV groups showed a significantly greater decrease in HBV DNA levels over time, and a significantly higher SVR compared to patients receiving Peg-INFα-2a monotherapy (both *P* values <0.05). Although patients receiving combination therapy had a significantly higher change in serum HBsAg levels compared to the monotherapy group, there was no significant difference in liver HBsAg levels between the three treatment groups.

**Conclusion:**

This study demonstrated that in patients with a poor virological response after 12 weeks of treatment with Peg-IFNα-2a alone, addition of ADV or ETV significantly reduced HBV DNA levels, serum HBsAg levels, and increased SVR. Individualization of anti-viral therapy would ensure that only patients who do not respond to Peg-IFNα-2a would receive combination therapy. Our data have important implications for the treatment of CHB patients who fail to show an early response to Peg-IFNα-2a monotherapy.

**Trial registration:**

This trial was retrospectively registered on 2012 May 24 at the China Clinical Trials Registry (ChiCTR-OCC-12002196).

## Background

It is estimated that approximately 350 million people worldwide are chronically infected with the hepatitis B virus (HBV) [1], and a large percentage of these patients have been shown to be at increased risk of developing liver cirrhosis or hepatocellular carcinoma (HCC) [2, 3]. The primary therapeutic goals in patients with chronic hepatitis B (CHB) are clearance of the hepatitis B surface antigen (HBsAg) from the serum, which is associated with normalization of ALT and histological improvement of inflammation and fibrosis [4, 5].

Currently approved treatments for CHB include two categories of drugs: 1) immune modulators such as Peg-INFα-2a, and 2) nucleoside (lamivudine, entecavir and telbivudine) and nucleotide analogs (adefovir dipivoxil and tenofovir disoproxil fumarate), which suppress viral replication by selectively inhibiting the activity of HBV DNA polymerase. Although nucleos(t)ide analogues (NAs) are cheap and safe, their limited long-term efficacy result in prolonged treatment periods, particularly in patients with HBeAg-negative CHB. Extended use of NAs has been shown to be associated with the generation of drug-resistant mutations [6]. In contrast, the duration of interferon-based therapy is predetermined and finite. However, only 30% of HBeAg-negative CHB patients treated with Peg-INFα-2a achieve a sustained virologic response (SVR) off-treatment, and HBsAg seroconversion in seen in 3–4% of patients after 1 year of treatment [7]. Although it was recently shown that CHB patients treated with PEG-IFN had higher rates of HBeAg seroconversion and HBsAg seroclearance compared to those treated with entecavir (ETV) [8], interferons have been shown to be effective only in patients with low viral loads, when the ALT levels are >2–5 ULN, or in patients infected with HBV genotype A/B. Additionally, although the rate of HBV DNA loss with interferon-based therapy can range from 14%–58.6%, more than 50% of the patients show low levels of viral replication, especially those with a poor early response to Peg-IFN [9–12]. Other challenges with using interferon-based therapy include the need for parenteral administration, frequent clinical follow-ups and the side-effects.

There has been a recent focus on developing novel therapeutic strategies for patients with a poor response to Peg-INF-α-2a. Some studies have combined the immunomodulatory properties of Peg-IFN along with the direct anti-viral activity of NAs in an attempt to improve therapeutic efficacy in CHB patients. Patients treated with a combination of Peg-INFα and lamivudine (LAM) had a higher on-treatment virological response rate compared to patients treated with LAM monotherapy, but there was no improvement in post-therapy response rates [9, 10]. Similarly, although HBeAg-negative CHB patients treated with a combination of Peg-INF-α-2a and adefovir dipivoxil (ADV) had a more rapid on-treatment HBV DNA suppression compared to those treated with Peg-INF-α-2a monotherapy, this did not translate to higher rates of virological response after suspension of treatment [13]. A total of 17 CHB patients treated with a combination of Peg-INF-α-2a and ETV for 48 weeks and observed for an additional 24 weeks showed a significant decrease in serum HBV DNA [14].

The protocol of anti-viral therapy in these combination studies was not adjusted based on early response. Optimizing therapeutic regimens based on early virological response and individual characteristics would ensure that only patients who fail to achieve a good response with Peg-INF-α-2a monotherapy would receive combination therapy, thereby shortening the course of therapy, enhancing virological response, decreasing the economic burden and conserving medical resources [15, 16].

In this study, we aimed to investigate the influence of additional nucleoside analogues on the SVR and the improvement of liver histology in CHB patients who had a poor virological response to Peg-INFα-2a at the end of 12 weeks of therapy.

## Methods

### Patient recruitment

A total of 178 patients diagnosed with CHB were recruited from the Department of Gastroenterology at China–Japan Union Hospital between September 2005 and December 2010. CHB diagnosis was based on the “Guideline for the Prevention and Therapy of Chronic Hepatitis B in China” issued by the Infectious Diseases Branch of the Chinese Medical Association, Hepatological Diseases Branch of the Chinese Medical Association, and the Chinese Foundation for Hepatitis Prevention and Control [17]. The study was performed in accordance with Good Clinical Practice and the ethical principles of the Declaration of Helsinki (ChiCTR-OCC-12002196). The study protocol was approved by a central independent ethics committee of China-Japan Union Hospital of Jilin University. All patients provided written informed consent.

Inclusion criteria were 1) HBV DNA ≥10^4^copies/ml (HBV DNA ≥ 300 copies /ml in the case of CHB patients with hepatic cirrhosis), 2) ALT ≥ULN, but ≤10 × ULN (normal or increased ALT in the case of CHB patients with hepatic cirrhosis), 3) serum total bilirubin <2 × ULN, 4) liver histological examination showing Knodell HAI ≥4 or ≥ G2 inflammatory necrosis. Additionally, all study patients received anti-viral therapy with Peg-IFNα-2a. Exclusion criteria were 1) presence of decompensated cirrhosis, 2) history of mental illness, 3) presence of epilepsy, cancer or symptomatic heart diseases, 4) evidence of drug abuse or alcohol abuse, 5) concomitant infection with HCV, HDV or HIV. Breast-feeding and pregnant women were also excluded.

This study was approved by the Institutional Review Board of the hospital, and informed consent was obtained from all study participants.

### Therapeutic protocol

All study patients received a subcutaneous injection of Peg-IFNα-2a (180 μg) once weekly for 12 weeks. The therapeutic protocol was then adjusted based on virological response. A total of 138 patients had a poor virological response after 12 weeks of anti-viral therapy with Peg-IFNα-2a. After fully communication with patients,According to patients’ willingness,Study patients were divided into 3 groups: (1) Patients in Group 1 (control group; *n* = 43) were treated with Peg-IFNα-2a for 48 weeks and were followed for 48 weeks after therapy. (2) Patients in Group 2 (*n* = 49) were treated with and 0.5 mg of ETV once daily for 48 weeks and were followed for 48 weeks after therapy. (3) Patients in Group 3 (*n* = 46) were treated with Peg-IFNα-2a and 10 mg of ADV once daily for 48 weeks and were followed for 48 weeks after therapy. Liver histological examination was performed in 38 of these patients before therapy and after follow-up, in order to evaluate the improvement of liver histology.
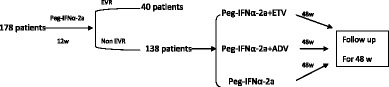


All study patients were subjected to a routine biochemical examination, routine urinalysis, and a routine blood test to evaluate thyroid function, and blood glucose levels. Patients were also evaluated for HBsAg levels, viral genotype. Routine blood tests were performed once weekly in the initial 4 weeks and thereafter once every 4 weeks. Biochemical examination and detection of HBV DNA and HBsAg levels were performed after 12 weeks of therapy, and once every 12 weeks thereafter. Patients receiving liver histological examination before therapy were evaluated for disease condition using the Knodell histology activity index (Knodell HAI) score, and were subjected to liver histological examination again after follow-up. Improvement in liver histology was defined as a reduction in Knodell score by ≥2 when compared to the baseline score, and no deterioration of hepatic fibrosis (deterioration of hepatic fibrosis is defined as an increase in Knodell fibrosis score by ≥1). Liver HBsAg levels were quantitated before and after anti-viral therapy in patients receiving liver histological examination.

### Evaluation of biochemical and virological markers

Viral markers were detected by ELISA (Shanghai Kehua Biotech Co., Ltd). Serum HBsAg levels were semi-quantitatively evaluated with the ElecsysIIdetection system using the S-P method according to the manufacturer’s instructions. Liver HBsAg expression was scored as 0, 1, 2, 3 and 4 based on cytoplasm and/or nuclear staining and as 25%, 25–50%, 50–75% and >75% based on the proportion of positive hepatocytes to total hepatocytes [18]. Liver function was detected with an automatic biochemical analyzer (Beckman, USA). HBV DNA levels in the serum and liver tissue were quantitated by real time fluorescence PCR using an RT-PCR kit (lower limit of detection is 300 copies/ml; COBAS TaqMan 48; Roche, Meylan, France). HBV genotypes were determined by PCR microplate hybridization ELISA (Guangzhou Lanxing Biotech Co., Ltd) according to the manufacturer’s instructions.

### Determination of therapeutic efficacy

Early virological response was defined as undetectable HBV DNA after anti-viral therapy for 12 weeks. After 12 weeks of therapy, patients were followed for 48 weeks. Sustained virological response (SVR) was defined as no change in therapeutic effectiveness after 6 months follow-up, and no recurrence. SVR was calculated as the ratio of patients with HBV DNA lower than the lower detection limit (<300copies/ml)to total patients.

Liver function was also re-examined regularly. However, it is important to note that patients treated with IFN and those with hepatic cirrhosis exhibit a transient increase in transaminases during anti-viral therapy with IFN. In addition, hepatic cirrhosis patients may occasionally exhibit a slight increase in transaminases although HBV DNA levels may be very low.

### Assessment of safety

Side effects such as fever, muscle ache, fatigue and leukopenia were evaluated. The dose of Peg-IFNα-2a dose was decreased when neutrophil counts reached ≤0.75 × 10^9^/L or platelet counts reached <50 × 10^9^/L; anti-viral therapy was stopped when the neutrophil count reduced to ≤0.5 × 10^9^/L or platelet count reduced to<30 × 10^9^/L.

### Determination of HBeAg conversion rate

HBeAg seroconversion rate was calculated as the ratio of HBeAg-positive patients who were HBeAb-positive after anti-viral therapy to the total number of HBeAg-positive patients.

### Statistical analysis

Patients’ demographic and baseline characteristic data were presented as mean ± standard deviations (SD) and median (IQR: 1st and 3rd quartiles) for continuous data with and without normal distribution; categorical data were presented as n (%). Differences among three groups were compared using one-way ANOVA and the Kruskall-Wallis test for continuous data with and without normal distribution, respectively; the Pearson Chi-square test was used for categorical ones.

The changes in HBV DNA levels after 4 wks, 12 wks, 24 wks, 36 wks, and 48 wks of treatment, and after 48 wks follow-up as compared with baseline HBV DNA levels were presented as mean ± SD at each time point for a given treatment group. Differences in HBV DNA levels over time were compared between the treatment groups using repeated measurement ANOVA test with post-hoc Bonferroni pair-wise comparisons.

The HBV DNA SVR and HBsAg conversion rate were represented as percentage of rate for each group, and the change in HBsAg levels was represented as mean with standard deviation (SD) for each group. Differences between the groups were compared using the Pearson Chi-square test for HBV DNA SVR and HBsAg conversion rate and one-way ANOVA with a post-hoc Bonferroni pair-wise comparisons for change in HBsAg levels.

The Knodell score (necroinflammatory score), Ishak score (fibrosis score) and improvement of liver histology among three groups were represented as median (IQR: 1st, 3rd quartiles) for Knodell score and Ishak score; and n (%) for improvement of liver histology. Differences between the groups were compared using the Kruskall-Wallis test for Knodell and Ishak scores. The improvement of liver histology was compared using Fisher’s exact test since some cell numbers were less than 5.

The association of the change in HBsAg levels with each treatment and the association of improvement rate with the change in HBsAg levels were evaluated. The change in HBsAg levels was represented as mean ± SD for each group and compared using one-way ANOVA with post-hoc Bonferroni pair-wise comparisons. The improvement rate was represented as percentages of patients for a given change in HBsAg levels. Differences between multiple changes in HBsAg levels were compared by Pearson Chi-square test.

All statistical assessments were two-tailed and *P* < 0.05 was considered significant. An adjusted significance level of 0.0167 (0.05/3) was considered for the post-hoc pair-wise comparison, Bonferroni test. All statistical analyses were performed using the SPSS statistics software (SPSS Inc., Chicago, IL, USA).

## Results

### Demographics and clinical characteristics

After the first 12 weeks of Peg-IFNα-2a treatment, a total of 43 study patients (24 males and 19 females) received Peg-IFNα-2a. Forty nine patients (35 males and 14 females) received Peg-IFNα-2a + ETV, and 46 patients (31 males and 15 females) received Peg-IFNα-2a + ADV. The mean ages of patients in the Peg-IFNα-2a, Peg-IFNα-2a + ETV, and Peg-IFNα-2a + ADV groups were 34.9 years (SD = 10.9), 37.4 years (SD = 9.5), and 38.6 years (SD = 10.0) respectively. There was no significant difference in the demographic data and baseline characteristics between the three groups (Table 1; all *P* values >0.05).

**Table 1:** Demographic and baseline characteristic data of study patients

Variables	Peg-IFNα-2a (*n* = 43)	Peg-IFNα-2a + ETV (*n* = 49)	Peg-IFNα-2a + ADV (*n* = 46)	*P* value
Age	34.86 ± 10.86	37.38 ± 9.51	38.63 ± 9.98	0.188
Sex, males (%)	24 (55.8%)	35 (71.4%)	31 (67.4%)	0.272
Baseline HBV DNA, (log_10_copies/mL)	6.47 ± 1.16	6.48 ± 1.01	6.24 ± 1.14	0.513
Baseline ALT, U/L	102 (87, 159)	119 (85, 162)	105 (87.8, 197.8)	0.744
HBeAg status				0.905
positive	28 (65.1%)	33 (67.3%)	32 (69.6%)	
negative	15 (34.9%)	16 (32.7%)	14 (30.4%)	
Genotype				0.183
B	10 (23.8%)	14 (28.6%)	19 (41.3%)	
C	32 (76.2%)	35 (71.4%)	27 (58.7%)	

### Efficacy of additional nucleoside analogues in patients treated with peg-IFNα-2a

**Fig. 1:**
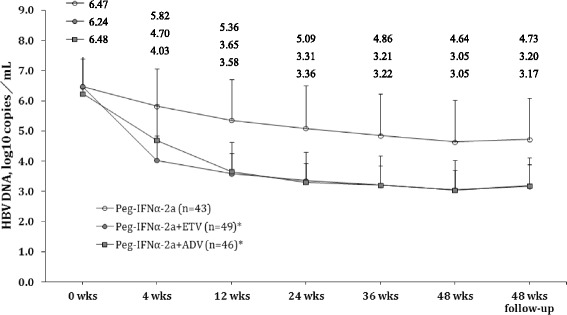
Dispersion of HBV DNA before and after treatment for each group. Data were represented as mean ± standard deviation (SD) for patients in Peg-IFNα-2a group, Peg-IFNα-2a + ETV group, and Peg-IFNα-2a + ADV group at baseline (0 wks), 4 wks, 12 wks, 24 wks, 36 wks, 48 wks of therapy, and 48 wks follow-up. Abbreviations: wks, weeks; ETV, Entecavir, 0.5 mg; ADV, Adefovir 10 mg. *P < 0.0167, indicates a significant difference compared to the Peg-IFNα-2a group. There was no significant difference between the Peg-IFNα-2a + ETV and Peg-IFNα-2a + ADV groups

The temporal profiles of HBV DNA levels were evaluated before and after treatment (Fig. 1). Patients in the Peg-INFα-2a + ETV as well as the Peg-INFα-2a + ADV groups showed a significantly greater decrease in HBV DNA levels over time compared to the group treated with Peg-INFα-2a alone (both *P* values <0.0167). Notably, Peg-INFα-2a + ETV and Peg-INFα-2a + ADV had similar efficacies, resulting in overlapping profiles after week 12 of treatment, but none patient achieve HBsAg loss during or after therapy (Fig. 1).

**Fig. 2:**
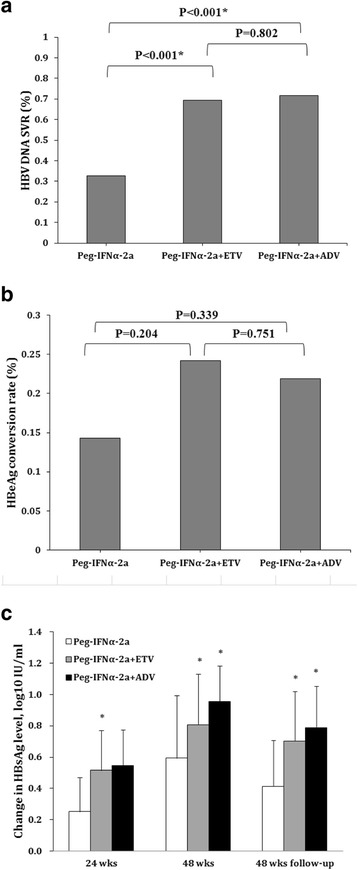
Summary of HBV DNA SVR (**a**), HBeAg conversion rate (**b**), and change in serum HBsAg levels after treatments (**c**) by groups. The change in HBsAg levels after therapy was calculated as the difference between baseline HBsAg level and the HBsAg level at a specific time point. HBV DNA SVR was represented as percentage of rate and the change in HBsAg levels was represented as mean with standard deviation (SD).* *P* < 0.001, indicates a significant difference

There was a significant difference in SVR between the three treatment groups (Fig. 2a). Patients in the Peg-IFNα-2a + ETV and Peg-IFNα-2a + ADV groups had significantly higher SVRs(69.4%, and 71.7%) compared to the group treated with Peg-IFNα-2a alone (32.6%), both *P* values <0.001.

Patients in the Peg-IFNα-2a + ETV and Peg-IFNα-2a + ADV groups had higher HBeAg conversion rates compared to the group treated with Peg-IFNα-2a alone, although these differences were not significant (Peg-IFNα-2a vs. Peg-IFNα-2a + ETV: 14.3% vs. 24.2%, *P* = 0.204; Peg-IFNα-2a vs. Peg-IFNα-2a + ADV: 14.3% vs. 21.9%, *P* = 0.339) (Fig. 2b).

The changes in serum HBsAg levels compared to basal levels were evaluated at week 24 and week 48 of treatment and at 48 weeks after treatment. At each time point, patients in the Peg-IFNα-2a + ETV and Peg-IFNα-2a + ADV groups exhibited significantly higher reduction in HBsAg levels compared to patients treated with Peg-IFNα-2a alone (both *P* values <0.0167; Fig. 2c).

Thirty eight patients (11 in Peg-IFNα-2a group, 14 in Peg-IFNα-2a + ETV group, and 13 in Peg-IFNα-2a + ADV group) received liver histological examinations before therapy and after the 48 week follow-up period. (Liver histological examination was performed in a fraction of the study patients since not all study patients were willing to undergo this procedure). There was no significant difference in any of these indices between the three groups (All P >0.05). There was no significant difference in improvement of liver histology between the three groups (27.3%, 64.3% and 69.2% for Peg-IFNα-2a, Peg-IFNα-2a + ETV and Peg-IFNα-2a + ADV groups, respectively; *P* > 0.05) (Table 2).

**Table 2:** Knodell and Ishak scores before and after anti-viral therapy and improvement of liver histology after anti-viral therapy in the three treatment groups (*N* = 38)

Variables	Peg-IFNα-2a (*n* = 11)	Peg-IFNα-2a + ETV (*n* = 14)	Peg-IFNα-2a + ADV (*n* = 13)	*P* value
Before anti-viral therapy				
Ishak score	3 (2, 3)	3 (2, 4)	4 (3, 4)	0.070
Knodell score	6 (5, 7)	6 (4.7, 7)	6 (5, 7.5)	0.154
After anti-viral therapy				
Ishak score	3 (2, 3)	2.5 (2, 3.3)a	3 (2.5, 3.5)a	0.613
Knodell score	4 (4, 5)b	4 (3, 5)b	4 (3, 6)b	0.573
Improvement of liver histology	3 (27.3%)	9 (64.3%)	9 (69.2%)	0.083

Data were represented as median (IQR: 1st, 3rd quartiles) for Knodell score and Ishak score; and n (%) for improvement of liver histology

Differences between groups were compared using the Kruskall-Wallis test for Knodell and Ishak scores; Differences between pre- and post-inflammation were compared using the Wilcoxon Sign-rank test because the Knodell score and Ishak score were ordinal data. The improvement of liver histology was compared using Fisher’s exact test since some cell numbers were less than 5

There was no significant difference between the groups

ab *P* < 0.05, significant difference compared with pre-treated a (Ishak score) and b (Knodell score) for a given group

The change in liver HBsAg levels was evaluated after 48 weeks of treatment in the 19 HBsAg-positive patients who received liver histological examination (5 in the Peg-IFNα-2a, 8 in Peg-IFNα-2a + ETV, and 6 in Peg-IFNα-2a + ADV. There was no significant difference (*P* > 0.05) in HBsAg levels between the three groups after 48 weeks of treatment (mean of 0.4 (SD = 0.55) for the Peg-IFNα-2a group, 1.4 (SD = 1.06) for the Peg-IFNα-2a + ETV group, and 1.8 (SD = 1.2) for the Peg-IFNα-2a + ADV group).

The rate of improvement versus the change in HBsAg levels was calculated in the 19 HBsAg-positive patients who received liver histological examination. The rate of improvement was 100% (3/3) in patients who had a change in HBsAg level = 3. The rate of improvement was 60% (3/5) in patients who had a change in HBsAg level = 2; 40% (2/5) in patients who had a change in HBsAg level = 1, and 50% (3/6) in patients who had a change in HBsAg level = 0. There was no significant association between rate of improvement versus the change in HBsAg levels (*P* = 0.459).

All study patients were monitored for adverse events. The most common adverse events reported were fatigue and muscle ache, fever, and thrombocytopenia (Table 3).

**Table 3:** Summary of adverse events

Adverse events	Peg-IFNα-2a (*n* = 43)	Combination groups (*n* = 95)
Fatigue and muscle ache	36 (83.7%)	76 (80%)
Fever	30 (70%)	70 (73.7%)
Thrombocytopenia	22 (51.2%)	47 (49.5%)
Anemia	16 (37.2%)	36 (37.9%)
Neutropenia	14 (32.6%)	33 (34.7%)
Loss of appetite	15 (34.9%)	30 (31.6%)
Hair Loss	10 (23.3%)	24 (25.3%)
Abnormal blood glucose	2 (4.7%)	5 (5.3%)
Rash	2 (4.7%)	3 (3.2%)
Hypothyroidism	1 (2.3%)	1 (1.1)

Data were summarized as n (%) for given adverse events in each group

## Discussion

In this study, patients who had a poor virological response after 12 weeks of Peg-IFNα-2a monotherapy were treated for an additional 48 weeks with a combination of Peg-IFNα-2a + ETV or Peg-IFNα-2a + ADV. Both regimens of combination therapy resulted in a significantly greater decrease in HBV DNA levels over time, and a significantly higher SVR compared to Peg-INFα-2a monotherapy. Although patients receiving combination therapy had a significantly higher change in serum HBsAg levels compared to the monotherapy group, there was no significant difference in liver HBsAg levels between the three treatment groups.

A number of studies have investigated therapeutic strategies which combined the immunomodulatory effects of Peg-IFNα-2a along with the direct anti-viral activity of NAs for patients with a poor response to anti-viral therapy. The addition of LAM to Peg-IFN therapy failed to enhance therapeutic efficacy compared to monotherapy in early studies [9, 10, 19]. However, a recent meta-analysis suggested that a combination of PEG-IFN and LAM increased SVR [20]. A combination of Peg-IFN with ADV or ETV for 48 weeks had a significantly higher therapeutic efficacy in HBeAg-negative CHB patients compared to Peg-IFN monotherapy [14, 21]. A recent study showed the efficacy of a sequential treatment strategy where patients received 24 weeks of telbuvidine followed by 24 weeks of Peg-IFNα-2a [22]. It is important to note that these studies used combination therapy as the first-line therapy, whereby patients having the potential to achieve a virological response with Peg-IFN alone also received combination therapy. In the present study, 138 study patients were treated Peg-IFNα-2a for 12 weeks, and only 95 patients who had a poor virological response at the end of this period received combination therapy of Peg-IFNα-2a with either ETV or ADV for 48 weeks. The Peg-IFN-α2a + ETV and Peg-IFN-α2a + ADV groups showed a reduction in HBV DNA by 3.3 and 3.0 log_10_ copies/mL, respectively and the proportion of patients with undetectable HBV DNA was 69.4% and 71.7%, respectively, which were significantly higher than that in patients treated with Peg-IFNα-2a alone.

Anti-viral therapy with Peg-IFN was shown to improve liver histology in 38–48% of patients and significantly delay disease progression [23]. A meta-analysis of 26 prospective studies also revealed that reduction of HBV DNA levels after anti-viral therapy was positively related to improvement of liver histology [24]. In the present study, a significantly higher percentage of patients in the Peg-IFN-α2a + ADV and Peg-IFN-α2a + ETV groups showed an improvement in liver histology compared to the Peg-IFNα-2a monotherapy group (69.2% and 64.3% vs. 27.3%). These data suggested that in patients with an early poor virological response to Peg-IFN-α2a, addition of NAs could promote inhibition of viral replication, which is associated with SVR and improvement of liver histology. These findings have important implications for attenuation of hepatic inflammation and fibrosis and slowing the progression of hepatic cirrhosis in CHB patients.

Baseline and on-treatment HBsAg levels have been shown to be strong predictors of therapeutic efficacy, and patients with HBsAg levels >20,000 IU/ml after 24 weeks of Peg-IFNα-2a treatment failed to achieve a virologic response [25]. Reduction in HBsAg levels has been shown to be closely related to seroconversion of HBeAg and HBsAg, and a larger reduction in HBsAg levels during therapy is associated with a higher possibility of HBsAg loss after long-term anti-viral therapy [26–29]. However, the association between serum HBsAg levels and liver histological score and HBV DNA levels remains unclear. One study reported that serum HBsAg levels were negatively associated with HBV DNA levels [30], while another study showed that 1) HBV DNA in the liver of CHB patients and HBV cccDNA before therapy were positively related to serum HBsAg, and 2) the reduction in serum HBsAg was positively associated with the reduction in HBV DNA in the liver and HBV cccDNA after combination therapy with LAM and Peg-IFN [31, 32]. In the present study, we showed that patients in the Peg-IFN-α-2a + ADV and Peg-IFN-α-2a + ETV groups had a more significant change in serum HBsAg levels compared to patients in the Peg-IFN-α-2a monotherapy group, as well as a higher rate of HBsAg seroconversion. Our data suggested that using combination therapy to achieve modulation of the immune response by Peg-IFNα-2a and inhibition of viral inhibition by Peg-IFNα-2a and NAs, significantly enhanced therapeutic efficacy which was reflected by a reduction in serum HBsAg. However, there was no correlation between change in liver HBsAg levels and rate of improvement in liver histology. The current study about the relation of serum HBsAg and liver histologic is not very clear, all the results are also different. This study not only observed the changes of serum HBsAg levels, also for the iver histologic HBsAg levels, designed to examine whether changes in the level of HBsAg can predict the effect. But because the patients with liver histological examination is less, the results found no significant differences.

We aimed to investigate the influence of additional nucleoside analogues on the SVR and the improvement of liver histology in CHB patients who had a poor virological response to Peg-INFα-2a at the end of 12 weeks of therapy. We can further observe the effect of sequential interferon therapy following nucleoside analogue for a long time .

## Conclusions

In summary, this study demonstrated that in patients with a poor virological response after 12 weeks of treatment with Peg-IFNα-2a alone, addition of ADV or ETV significantly reduced HBV DNA levels, serum HBsAg levels, and increased SVR. Individualization of anti-viral therapy would ensure that only patients who do not respond to Peg-IFNα-2a would receive combination therapy. Our data have important implications for the treatment of CHB patients who fail to show an early response to Peg-IFNα-2a monotherapy.

## Abbreviations

ADV: adefovir dipivoxil
cccDNA: covalently closed circular HBV DNA
CHB: chronic hepatitis B
ETV: entecavir
HBV: hepatitis B virus
HCC: hepatocellular carcinoma
LAM: lamivudine
PEG-IFN-α-2a: pegylated interferon alpha 2a
SVR: sustained virologic response
